# A complex interaction between Wnt signaling and TNF-α in nucleus pulposus cells

**DOI:** 10.1186/ar4379

**Published:** 2013-11-14

**Authors:** Akihiko Hiyama, Katsuya Yokoyama, Tadashi Nukaga, Daisuke Sakai, Joji Mochida

**Affiliations:** 1 Department of Orthopaedic Surgery, Surgical Science, Tokai University School of Medicine, 143 Shimokasuya, Isehara, Kanagawa 259-1193, Japan; 2 Research Center for Regenerative Medicine, Tokai University School of Medicine, 143 Shimokasuya, Isehara, Kanagawa 259-1193, Japan

## Abstract

**Introduction:**

Increased expression of the proinflammatory cytokine TNF-α in intervertebral discs (IVDs) leads to inflammation, which results in progressive IVD degeneration. We have previously reported that activation of Wnt-β-catenin (hereafter called Wnt) signaling suppresses the proliferation of nucleus pulposus cells and induces cell senescence, suggesting that Wnt signaling triggers the process of degeneration of the IVD. However, it is not known whether cross talk between TNF-α and Wnt signaling plays a role in the regulation of nucleus pulposus cells. The goal of the present study was to examine the effect of the interaction between Wnt signaling and the proinflammatory cytokine TNF-α in nucleus pulposus cells.

**Methods:**

Cells isolated from rat nucleus pulposus regions of IVDs were cultured in monolayers, and the expression and promoter activity of Wnt signaling and TNF-α were evaluated. We also examined whether the inhibition of Wnt signaling using cotransfection with Dickkopf (DKK) isoforms and Sclerostin (SOST) could block the effects of pathological TNF-α expression in nucleus pulposus cells.

**Results:**

TNF-α stimulated the expression and promoter activity of Wnt signaling in nucleus pulposus cells. In addition, the activation of Wnt signaling by 6-bromoindirubin-3′-oxime (BIO), which is a selective inhibitor of glycogen synthase kinase 3 (GSK-3) activity that activates Wnt signaling, increased TNF-α expression and promoter activity. Conversely, the suppression of TNF-α promoter activity using a β-catenin small interfering RNA was evident. Moreover, transfection with DKK-3, DKK-4, or SOST, which are inhibitors of Wnt signaling, blocked Wnt signaling-mediated TNF-α activation; these effects were not observed for DKK-1 or DKK-2.

**Conclusions:**

Here, we have demonstrated that Wnt signaling regulates TNF-α and that Wnt signaling and TNF-α form a positive-feedback loop in nucleus pulposus cells. The results of the present study provide in vitro evidence that activation of Wnt signaling upregulates the TNF-α expression and might cause the degeneration of nucleus pulposus cells. We speculate that blocking this pathway might protect nucleus pulposus cells against degeneration.

## Introduction

Wnt proteins are important intervertebral disc (IVD) cell regulatory factors. We have previously analyzed Wnt-β-catenin (hereafter called Wnt) signaling in nucleus pulposus cells and reported that activation of Wnt signaling suppresses the proliferation of nucleus pulposus cells and induces cell senescence, suggesting that Wnt signaling triggers the process of degeneration of IVDs [1-3]. Increased expression of both matrix metalloproteinase (MMP)13 and a disintegrin and metalloproteinase with thrombospondin motifs (ADAMTS)-5 was also reported recently in the IVDs of β-catenin knockout mice, which is consistent with the observed IVD degeneration. Moreover, those authors found that IVD degeneration was suppressed when an inhibitor of MMP13 was administered to β-catenin knockout mice. These results led to the conclusions that β-catenin is a key factor that is responsible for the maintenance of the IVD tissue structure [4]. To date, at least three intracellular signaling pathways have been shown to mediate Wnt signaling: the Wnt/β-catenin pathway, the Wnt/Ca^2+^ pathway, and the planar cell polarity (PCP) pathway [5,6]. As the signaling pathways that play crucial roles during embryogenesis are tightly regulated, the expression of Wnt proteins and Wnt antagonists is exquisitely restricted, both temporally and spatially, during development [7]. Wnt signaling is activated upon binding of several members of the Wnt protein family to the Frizzled/low-density lipoprotein receptor-related protein 5 or 6 (Fz-LRP5/6) receptor complex. This causes β-catenin stabilization and translocation to the nucleus, where it binds to the lymphoid enhancer factor and T-cell factor (LEF and TCF) transcription factors to activate Wnt target gene expression [8-11]. However, the upstream and downstream regulatory elements of Wnt signaling in IVD cells remain unknown, and the molecular mediators in the IVD are poorly understood.

Low back pain is strongly associated with IVD degeneration, which in turn is associated with sciatica and disc herniation [12,13]. The IVD consists of the peripheral annulus fibrosus that encloses a gel-like tissue, the nucleus pulposus. During development, the highly hydrated nucleus pulposus is populated by clusters of large vacuolated notochordal cells of distinct molecular phenotype. In humans and some other species (for example, cattle and chondrodystrophoid dogs), notochordal cells disappear before maturity, to be replaced by chondrocyte-like cells [14,15]. IVD degeneration, which is linked to persistent back pain, is characterized by profound anatomical and biological changes that include a decrease in cell number and a simultaneous increase in the expression of catabolic cytokines. Elevated levels of proinflammatory cytokines and other inflammatory mediators have been reported to be present in degenerate IVDs, including TNF-α, IL-1β, IL-6, and prostaglandin E2 (PGE2) [16,17]. These cytokines upregulate MMPs and ADAMTs gene expression, and downregulate SOX-9, type II collagen, and aggrecan expression in articular chondrocytes [18,19]. During IVD degeneration and IVD herniation, nucleus pulposus and annulus fibrosus cells produce high levels of proinflammatory cytokines. Moreover, both TNF-α and IL-1β stimulate the production of nerve growth factor (NGF), brain-derived neurotrophic factor (BDNF), and vascular endothelial growth factor (VEGF), which are molecules that are associated with the nerve ingrowth and angiogenesis observed in nucleus pulposus cells [20]. Increased TNF-α levels in IVD lead to inflammation and apoptosis, which results in progressive IVD degeneration. Currently, it is not known whether cross-talk between TNF-α and Wnt signaling plays a role in the regulation of nucleus pulposus cells. Therefore, the goal of the present study was to examine the effect of the interaction between Wnt signaling and the proinflammatory cytokine TNF-α in nucleus pulposus cells. Here, we showed that Wnt signaling regulated TNF-α and that Wnt signaling and TNF-α form a positive-feedback loop in nucleus pulposus cells.

## Methods

Animal experiments were performed according to a protocol approved by the Animal Experimentation Committee of our institution (The Institutional Animal Care and Use Committee at Tokai University, approved number: 135011).

### Reagents and plasmids

To determine the β-catenin-TCF/LEF transcriptional activity, nucleus pulposus cells and annulus fibrosus cells were transiently transfected with the TCF/LEF reporter gene Topflash (optimal TCF binding site) or Fopflash (mutated TCF binding site) (Upstate Biotechnology, Lake Placid, NY, USA). The Fopflash construct is identical to the Topflash construct, with the exception that it contains mutated copies of TCF/LEF binding sites and is used as a control to measure nonspecific activation of the reporter construct. K3-luc (TNF-α promoter element; number 11110) and the SOST expression plasmid (number 10842) were purchased from Addgene (Cambridge, MA, USA). The wild-type (WT)-β-catenin expression plasmid and the backbone plasmid were provided by Dr Raymond Poon (Hospital for Sick Children, University of Toronto, Toronto, Ontario, Canada). The β-catenin small interfering RNA (siRNA) (number sc-29209) and control siRNA duplexes were purchased from Santa Cruz Biotechnology (Santa Cruz, CA, USA). The Dickkopf (DKK)-1, -2, -3, and -4 expression plasmids and the backbone (pMY-IRES-EGFP) plasmids were provided by Dr Siegfried Janz (Laboratory of Genetics, Center for Cancer Research, National Cancer Institute, National Institutes of Health, Bethesda, MD, USA) [21].

We used the vector pGL4.74 (Promega, Madison, CA, USA) containing the *Renilla reniformis* luciferase gene as an internal transfection control. Recombinant TNF-α was purchased from (210-TA, R & D Systems, Abingdon, UK). We used 6-bromoindirubin-3′-oxime (BIO) (number 361550; Calbiochem, San Diego, CA, USA) to examine Wnt signaling activity. BIO is a cell-permeable, highly potent, selective, reversible, and ATP-competitive specific inhibitor of glycogen synthase kinase (GSK)-3a/b activity [22].

### Isolation of IVD cells

Nucleus pulposus cells and annulus fibrosus cells were isolated from the lumbar discs of 11-week-old Sprague Dawley rats (n = 32) using methods reported by Hiyama *et al*. [3]. The isolated cells were maintained in (DMEM) and 10% fetal bovine serum (FBS) supplemented with antibiotics at 37°C in a humidified atmosphere of 5% CO_2_. Confluent nucleus pulposus and annulus fibrosus cells were harvested and subcultured in 10-cm dishes. Low-passage (<3) cells cultured in monolayers were used for all experiments, because cells obtained from the rat IVD tissues exhibited variable morphology until passages 2 or 3.

### Immunofluorescence staining

Nucleus pulposus cells were plated in flat-bottom 96-well plates (3 × 10^3^ cells/well) and incubated for 24 h. The cells were treated with 10 ng/mL TNF-α or 1.0 μM BIO, fixed with 4% paraformaldehyde, permeabilized with 0.5% Triton X-100 (vol/vol) in PBS, blocked with PBS containing 10% FBS, and incubated overnight at 4°C with antibodies against TNF-α (sc-1350, 1:100 dilution; Santa Cruz Biotechnology) or β-catenin (1:200 dilution; Cell Signaling Technology, Danvers, MA, USA). The cells were washed and incubated with anti-rabbit Alexa Fluor 488 secondary (green) antibodies (Invitrogen, Carlsbad, CA, USA) at a dilution of 1:200 and 10 μM 4′,6-diamidino-2-phenylindole (DAPI) for 1 h at room temperature, for nuclear staining. The samples were observed under a fluorescence microscope connected to a digital imaging system. Negative controls without the primary antibody were prepared.

### Real-time reverse transcription polymerase chain reaction (RT-PCR) analysis

Nucleus pulposus cells were cultured in 10-cm plates (5 × 10^5^ cells/plate) with or without TNF-α for 24 h, and the total RNA was extracted from the cells using the TRIzol RNA isolation protocol (Invitrogen). RNA was treated with RNase-free DNAse I. Total RNA (100 ng) was used as a template for the real-time PCR analyses. The cDNA was synthesized via the reverse transcription of mRNA, as described previously [3]. Reactions were set up in triplicate in 96-well plates using 1 μL of cDNA with SYBR Green PCR Master Mix (Applied Biosystems, Carlsbad, CA, USA), to which gene-specific forward and reverse PCR primers for *Wnt1*, *Wnt3a*, *Wnt4*, *Wnt5a*, *Wnt5b*, *Wnt6*, *Wnt10a*, *Wnt10b*, *Wnt16*, *LRP5*, *LRP6*, *β-catenin*, *LEF1*, *TCF4*, *TNF-α*, *TNFR1* and *TNFR2* were added. The primers were synthesized by (Takara Bio Inc, Otsu, Shiga, Japan) or (FASMAC Corp, Atsugi, Kanagawa, Japan) and are shown in Table 1. PCR reactions were performed in an Applied Biosystems 7500 Fast system, according to the manufacturer’s instructions. A control gene, *glyceraldehyde 3-phosphate dehydrogenase* (*GAPDH*), was used to normalize each sample, and the arbitrary intensity threshold (C_t_) of amplification was computed. The expression scores were obtained using the ΔΔC_t_ calculation method.

**Table 1:** Primers for real-time PCR

** Target**	** NCBI number**	** Forward primer, 5'-3'**	** Reverse primer, 5'-3'**
β-catenin	AF_121265.1	GCCAGTGGATTCCGTACTGT	GAGCTTGCTTTCCTGATTGC
Wnt1	NM_001105714.1	TCCTCATGAACCTTCACAATAACGA	TTGCACTCTTGGCGCATCTC
Wnt3a	NM_001105715.1	TGTGAGGTGAAGACCTGCTG	AAAGTTGGGGGAGTTCTCGT
Wnt4	NM_053402.1	GAAACGTGCGAGAAGCTCAAAG	AAAGGACTGTGAGAAGGCTACG
Wnt5a	NM_022631.1	TGCCACTTGTATCAGGACCA	TGTCTCTCGGCTGCCTATTT
Wnt5b	NM_001100489.1	TGACTACTGCCTGCGAAATG	AAAGCAACACCAGTGGAACC
Wnt6	NM_001108226.1	GTCGACTTTGGGGATGAGAA	AAAGCCCATGGCACTTACAC
Wnt10a	NM_001108227.1	GTTCCTAGCTCAGGCAGGTG	AAGTCTGTGGAGGGGGAGAT
Wnt10b	NM_001108111.1	CTGGTGCTGTTACGTGCTGT	GGAGCCATGATTAACCGAAA
Wnt16	NM_001109223.1	TGGCTGTAACCTCCTCTGCT	GAGGCAATCTCATGCTAGGC
LRP5	NM_001106321.2	CATCCATGCTGTGGAGGA	TGTCTCGGGCACAAGGAT
LRP6	NM_001107892.1	CATGATACGAAGGCACAAGAA	TCTGATTTGGAACCGAGCTT
LEF1	NM_130429.1	TGGTAAACGAGTCCGAAATCA	TGTGTTTGTCCGACCACCT
TCF4	NM_053369.1	TCCAACCCTTCAACTCCTGT	CGTTTCGAGACCAAACAGC
TNF-α	NM_012675.3	TGAACTTCGGGGTGATCG	GGGCTTGTCACTCGAGTTTT
TNFR1	NM_013091.1	AATGAGTGCACCCCTTGC	CCTGGGGGTTTGTGACATT
TNFR2	NM_130426.4	GAGGCCCAAGGGTCTCAG	GCTGCCATGGGAAGAATC

Transcript and sequence of each primer used in the real-time RT-PCR experiments.

### Gene-suppression studies using siRNA

We silenced β-catenin expression in nucleus pulposus cells by using siRNA technology. In brief, nucleus pulposus cells were transferred into 24-well plates at a density of 6 × 10^4^ cells/well 1 day before transfection. The next day, cells were treated with β-catenin siRNA or control siRNA duplexes at a final concentration of 100 to 500 ng using Lipofectamine 2000. Cells also received TNF-α promoter constructs and the pGL4.74 plasmid at the time of transfection. Six hours after transfection, the medium was replaced with complete growth medium and the cells were allowed to recover for 18 h. Cells were then cultured for 24 h and luciferase activity was measured.

### Western blot analysis

Treated nucleus pulposus cells were placed immediately on ice and washed with cold PBS. Proteins were prepared using the CellLytic NuCLEAR extraction kit (Sigma-Aldrich, St Louis, MO, USA). All of the wash buffers and the final resuspension buffer included 1 × protease inhibitor cocktail (Roche, Basel, Switzerland), NaF (5 mM), and Na_3_VO_4_ (200 μM). Nuclear or total cellular proteins were separated on a sodium dodecyl sulfate (SDS) polyacrylamide gel and were electrotransferred onto nitrocellulose membranes (Bio-Rad, Hercules, CA USA). The membranes were blocked with 5% BSA in Tris-buffered saline and Tween 20 (TBST) (50 mM Tris, pH 7.6, 150 mM NaCl, and 0.1% Tween-20) and incubated overnight at 4°C in 5% BSA in TBST with anti-TNF-α (number sc-1350. 1:200; Santa Cruz Biotechnology) or anti-β-catenin (1:1,000 dilution; Cell Signaling Technology) antibodies. Immunolabeling was detected with an ECL reagent (Amersham Bioscience, Little Chalfont, UK). The Western blot data were quantified using Image J pixel analysis (NIH Image software). Data from the western blots were presented as band density normalized to that of the loading control (actin).

### Transfections and dual-luciferase™ assay

Nucleus pulposus cells and annulus fibrosus cells were transferred to 24-well plates at a density of 3 × 10^4^ cells/well 1 day before transfection. Cells were co-transfected with 100 to 500 ng of expression plasmids or the backbone vector together with the reporter plasmids. Lipofectamine 2000 (Invitrogen) was used as the transfection reagent. Cells were cultured for 24 h and treated with a specific concentration of TNF-α or BIO. The cells were harvested 24 h after treatment and a Dual-Luciferase™ reporter assay system (Promega) was used for the sequential measurements of firefly and *Renilla* luciferase activities. The results were normalized regarding transfection efficiency and were expressed as a relative ratio of luciferase to pGL4.74 activities (denoted as relative activity). Nucleus pulposus cells were transfected with a plasmid encoding green fluorescent protein, to check transfection efficiency, which was 60 to 70% in nucleus pulposus cells. The luciferase activities and relative ratios were quantified using a Turner Designs Luminometer Model TD-20/20 instrument (Promega).

### Statistical analyses

Typically, data were compiled from at least three independent triplicate experiments, each performed using separate cultures and on separate occasions. The responses were presented as the fold change relative to the untreated control. The data were presented as the mean ± SD. Data were compared between groups using Student’s *t*-test or analysis of variance, to assess variance. Significance was accepted at *P* <0.05 and is denoted with an asterisk in the figures.

## Results

### Effects of Wnt signaling on TNF-α expression in nucleus pulposus cells

**Figure 1:**
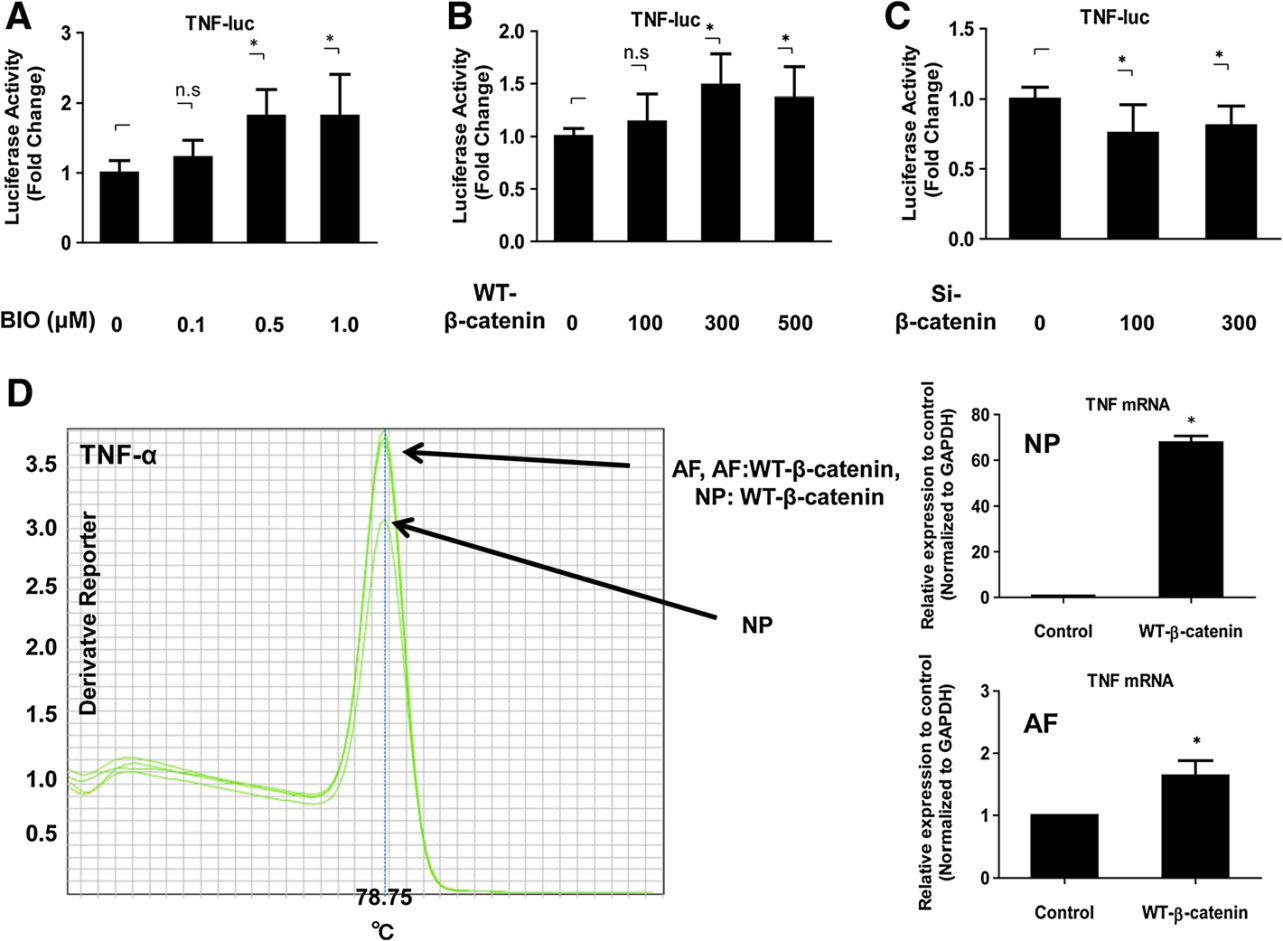
**Effect of Wnt signaling on TNF-α expression in nucleus pulposus cells. (A)** Nucleus pulposus cells transfected with the TNF-α reporter plasmid together with the pGL4.74 plasmid were treated with different concentrations of 6-bromoindirubin-3′-oxime (BIO) for 24 h. **(B** and **C)** Nucleus pulposus cells were co-transfected with the TNF-α reporter plasmid together with WT-β-catenin **(B)**, β-catenin siRNA (si-β-catenin) **(C)**, or empty vectors and the pGL4.74 vector. Cells were cultured for 24 h and luciferase reporter activity was measured. The results were normalized for transfection efficiency and are expressed as a relative ratio of luciferase to pGL4.74 activity (denoted as relative activity). **(D)** Real-time RT–PCR analysis of TNF-α mRNA levels in nucleus pulposus cells and annulus fibrosus cells transfected with the WT-β-catenin expression plasmid. Amplification of the TNF-α PCR products was verified by correct melting temperature. Error bars represent the SD. n.s., not significant; NP, nucleus pulposus; AF, annulus fibrosus.

Although Wnt signaling has been shown to play an important role in nucleus pulposus cells, interactions between Wnt signaling and TNF-α in these cells have not been described. Therefore, to study the relationship between Wnt signaling and TNF-α, initial experiments were performed to investigate the role of Wnt signaling in the transcriptional activity of TNF-α in nucleus pulposus cells. Cells treated with different concentrations of BIO exhibited an increase in the activity of the TNF-α promoter (BIO 0.1 μM, 1.22 ± 0.25, *P* = 0.2026; 0.5 μM, 1.81 ± 0.37, *P* <0.001; 1.0 μM, 1.81 ± 0.59, *P* <0.001) (Figure 1A). Nucleus pulposus cells were transiently transfected with plasmids encoding TNF-α and with a WT-β-catenin expression plasmid. Figure 1B shows that forced expression of WT-β-catenin significantly induced TNF-α promoter activity (WT-β-catenin 100 ng, 1.13 ± 0.26, *P* = 0.3801; 300 ng, 1.49 ± 0.30, *P* = 0.0076; 500 ng, 1.36 ± 0.30, *P* = 0.0304). To validate these findings, we performed loss-of-function experiments using an siRNA for β-catenin. Suppression of gene expression was confirmed by real-time RT-PCR (data not shown). TNF-α promoter activity was inhibited in nucleus pulposus cells that were co-transfected with the β-catenin siRNA (si-β-catenin 100 ng, 0.75 ± 0.20, *P* = 0.0041; 300 ng, 0.80 ± 0.14, *P* = 0.0244) (*P* <0.05) (Figure 1C). To confirm the reporter assay data, next we performed a real-time PCR analysis of *TNF-α* mRNA expression after transfection with the WT-β-catenin expression plasmid both in nucleus pulposus cells and annulus fibrosus cells. Figure 1D shows that nucleus pulposus cells and annulus fibrosus cells transfected with WT-β-catenin exhibited a significant increase in the gene expression of *TNF-α* compared with that observed in untransfected control cells. The expression of TNF-α was significantly higher in the nucleus pulposus than in the annulus fibrosus.

**Figure 2:**
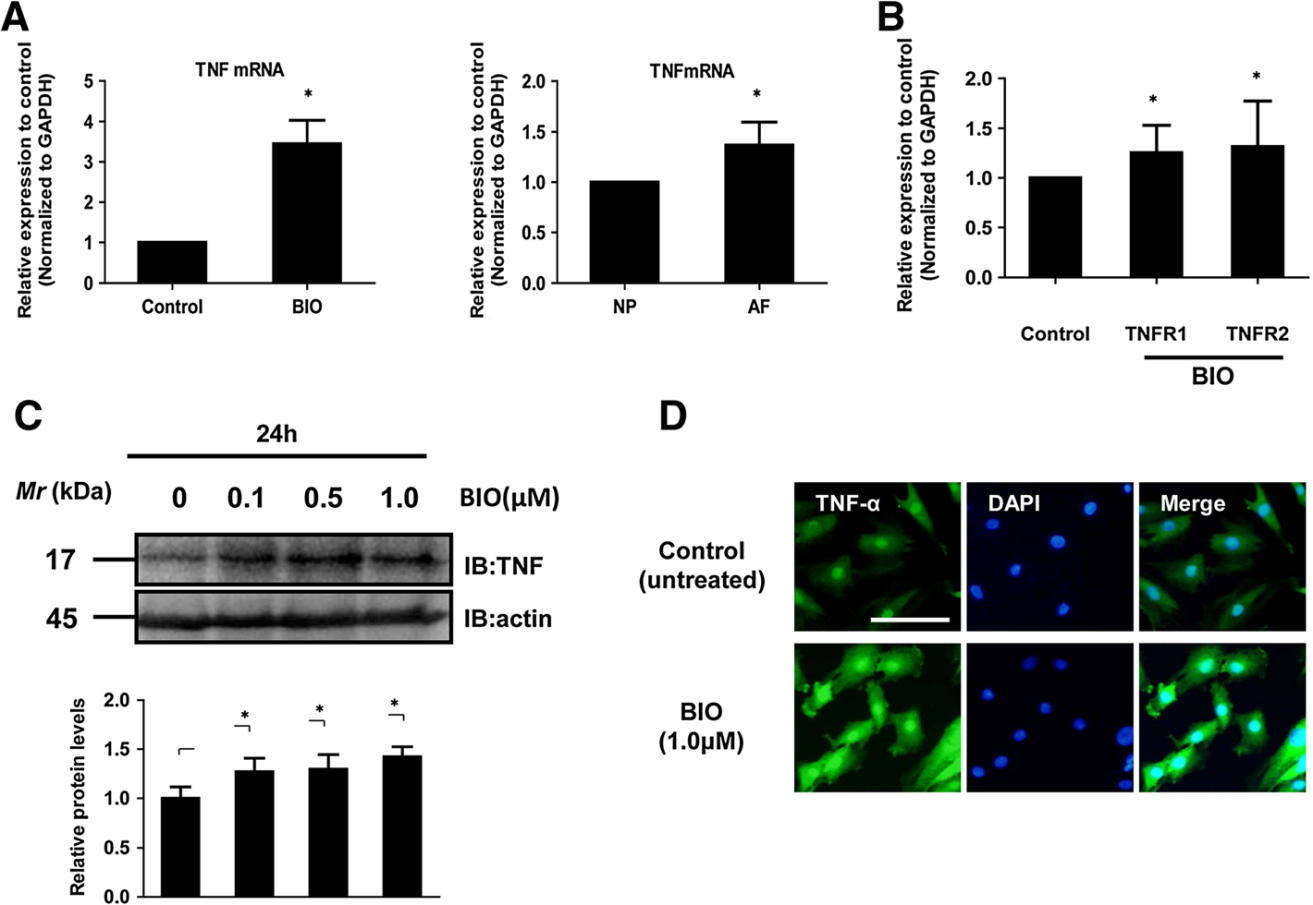
**Wnt signaling enhanced the expression of the *TNF*-α gene and protein in nucleus pulposus cells. (A)** TNF-α mRNA expression after exposure of nucleus pulposus cells to 6-bromoindirubin-3′-oxime (BIO) (1.0 μM) for 24 h, as assessed by real-time PCR (left panel). Real-time RT-PCR analysis of TNF-α mRNA levels in nucleus pulposus cells and annulus fibrosus cells (right panel). **(B)***TNFR1* mRNA and *TNFR2* mRNA expression after exposure of nucleus pulposus cells to BIO (1.0 μM) for 24 h, as assessed by real-time PCR. **(C)** Western blot analysis of TNF-α activation after treatment of nucleus pulposus cells with varying concentrations of BIO (0.0 to 1.0 μM). **(D)** Detection of TNF-α protein expression by immunofluorescence microscopy. Nucleus pulposus cells were cultured with or without 1.0 μM BIO for 24 h, fixed, and stained with an antibody against c-fos. Left: cells stained with an antibody to TNF-α; middle: cells stained with 4′,6-diamidino-2-phenylindole (DAPI), to identify healthy nuclei; right: cells stained with an antibody to TNF-α and with DAPI. Scale bar, 50 μm (original magnification, 20×).

**Figure 3:**
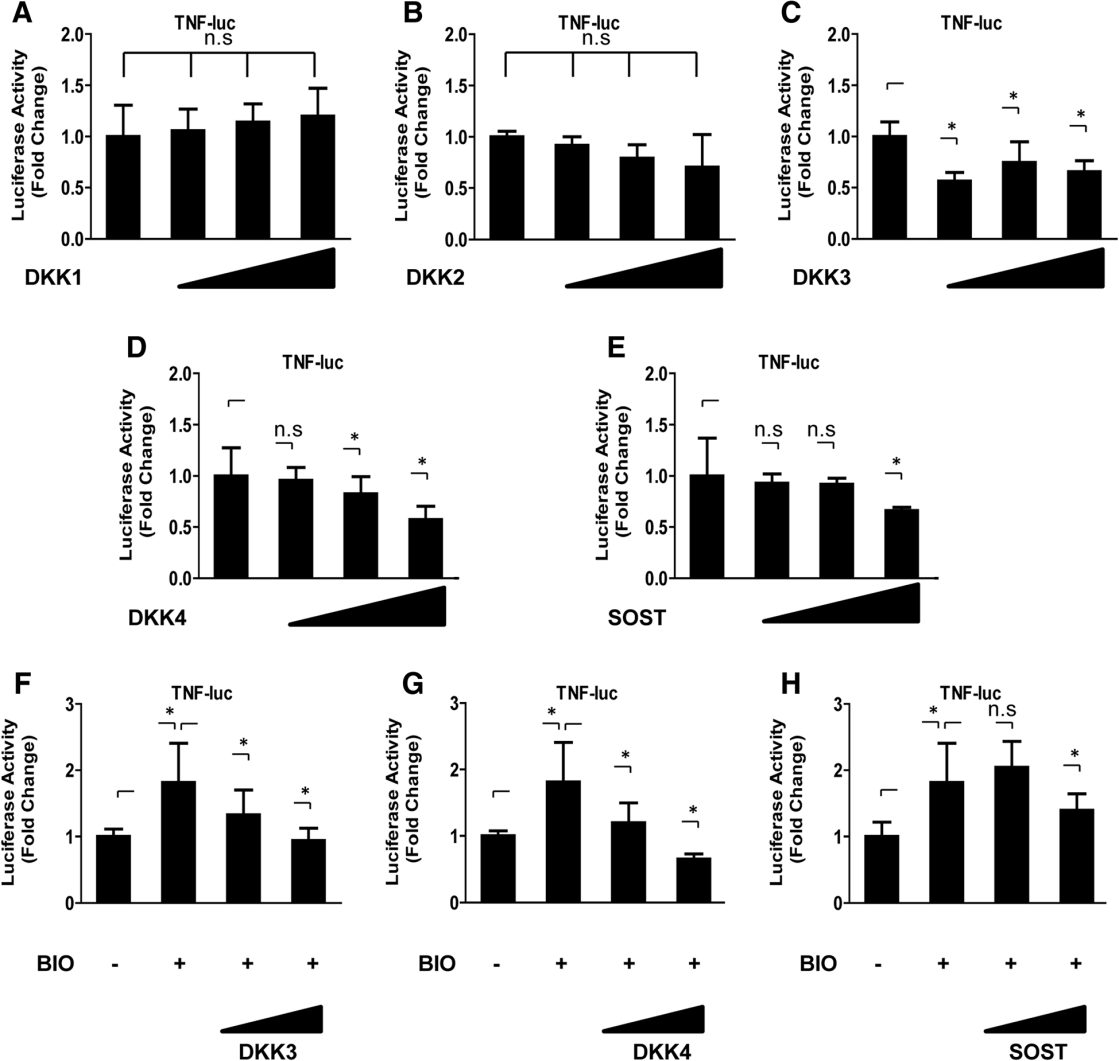
**Wnt signaling-induced upregulation of TNF-α was antagonized by DKK and Sclerostin (SOST). (A**–**E)** Nucleus pulposus cells were transfected with the TNF-α reporter construct and increasing concentrations of the WT-DKK plasmids (100 to 500 ng) or empty backbone plasmids **(A**: DKK-1, **B**: DKK-2, **C**: DKK-3, **D**: DKK-4, **E**: SOST). **(F**-**H)** The TNF-α reporter plasmid was transfected into nucleus pulposus cells together with the PGL4.74 vector, and was stimulated with 6-bromoindirubin-3′-oxime (BIO) in the presence or absence of DKK expression plasmids. Luciferase activity was measured 24 h after transfection **(F**: DKK-3, **G**: DKK-4, **H**: SOST). ^*^P <0.05 indicates significant differences between groups. n.s., not significant.

Real-time RT-PCR analysis also showed that activation of Wnt signaling by BIO increased the expression of *TNF-α* mRNA 3-fold compared with untreated cells (Figure 2A). Similarly, transfection of WT-β-catenin led to an increase in the expression of the *TNF-α* mRNA compared with untreated cells (data not shown). The expression of *TNF-α* mRNA was significantly higher (by 1.5-fold) in the annulus fibrosus than in the nucleus pulposus (*P* <0.01, Figure 2A). In addition, activation of Wnt signaling increased the expression of *TNFR1* mRNA and *TNFR2* mRNA compared with untreated cells (Figure 2B) (*TNFR1* mRNA, 1.25 ± 0.28; *TNFR2*, 1.32 ± 0.46) (*P* <0.05). To determine whether a concomitant elevation in TNF-α protein expression was associated with Wnt signaling, the cells were evaluated using western blotting (Figure 2C) and immunofluorescence analysis (Figure 2D). As shown in Figure 2C, immunoblotting of nucleus pulposus cells treated with BIO (1.0 μM, 24 h) showed an increased level of TNF-α protein compared with control nucleus pulposus cells. Similarly, immunofluorescence analysis using an anti-TNF-α antibody showed that BIO treatment (1.0 μM, 24 h) promoted the nuclear translocation of TNF-α more strongly in nucleus pulposus cells than in untreated cells. TNF-α was localized to the nucleus in cells treated with BIO (Figure 2D). To define a potential role of Wnt signaling on TNF in nucleus pulposus cells, we also used DKK-1, -2, -3, and -4 expression plasmids. No change in TNF-α promoter activity was observed when DKK-1 or DKK-2 plasmids were used (Figure 3A and B). Conversely, transfection with DKK-3 and DKK-4 led to a dose-dependent suppression in TNF-α promoter activity (Figure 3C and D). Similarly, the BIO-mediated induction of TNF-α promoter activity was suppressed by transfection with the DKK-3 or DKK-4 plasmids (Figure 3F and G). To validate these findings, we used a Sclerostin (SOST) expression plasmid and measured the activity of the TNF-α promoter in this condition; we detected a significant inhibition of TNF-α promoter at a concentration of 500 ng (Figure 3E and H).

### TNF-α enhanced Wnt transcriptional activity in nucleus pulposus cells

**Figure 4:**
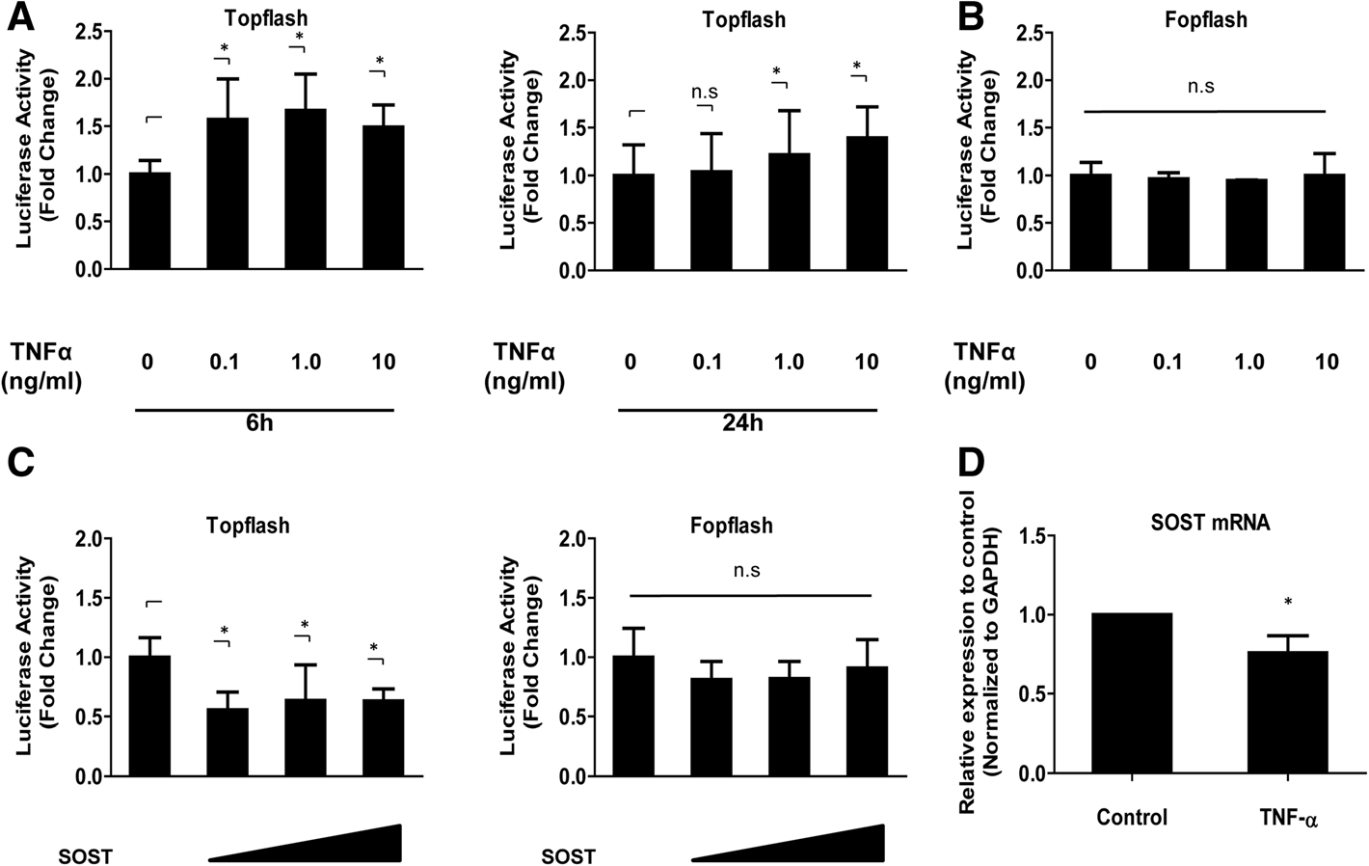
**Effect of TNF-α on Wnt signaling in nucleus pulposus cells. (A**-**B)** Cells transfected with the Topflash reporter plasmid **(A)** or the Fopflash plasmid **(B)** together with the pGL4.74 plasmid were treated with different concentrations of TNF-α for 6 to 12 h. **(C)** Nucleus pulposus cells were transfected with the Topflash reporter plasmid or the Fopflash reporter plasmid and increasing concentrations of the Sclerostin (SOST) plasmids (100 to 500 ng) or empty backbone plasmids. **(D)** SOST mRNA expression after exposure of nucleus pulposus cells to TNF-α (10 ng/mL) for 24 h, as assessed by real-time PCR. ^*^P <0.05 indicates significant differences between groups. Error bars represent the SD. n.s., not significant.

To investigate the role of the proinflammatory cytokine TNF-α in Wnt signaling in IVDs, we first examined the soluble TNF-α protein levels after the stimulation of TNF-α (10 ng/mL, 24 h) by western blotting. The results demonstrate that soluble TNF-α protein was significantly elevated after the stimulation of TNF-α compared to the control cells (Additional file 1). We next determined whether TNF-α induced Wnt transcriptional activity. We measured the activity of both Topflash (containing the WT TCF binding sites) and Fopflash (mutant Topflash) in nucleus pulposus cells after TNF-α treatment. After 6 to 24 h, we measured the activity of Topflash in nucleus pulposus cells. Figure 4A shows that there was a dose-dependent increase in the activity of Topflash upon TNF-α stimulation, whereas Fopflash activity was not affected by TNF-α treatment (Figure 4B). We then co-transfected nucleus pulposus cells with plasmids expressing SOST along with both Topflash and Fopflash reporter. Figure 4C shows that overexpression of SOST results in a decrease in the activity of Topflash, whereas overexpression of SOST has no effect on Fopflash reporter activity. To explore the premise that TNF-α regulates *SOST* mRNA expression, nucleus pulposus cells were treated with TNF-α and expression of *SOST* mRNA analyzed using real-time PCR. Figure 4D shows that treatment with TNF-α for 24 h significantly decreases *SOST* mRNA levels in nucleus pulposus cells.

### TNF-α enhanced Wnt related gene and protein expression in nucleus pulposus cells

**Figure 5:**
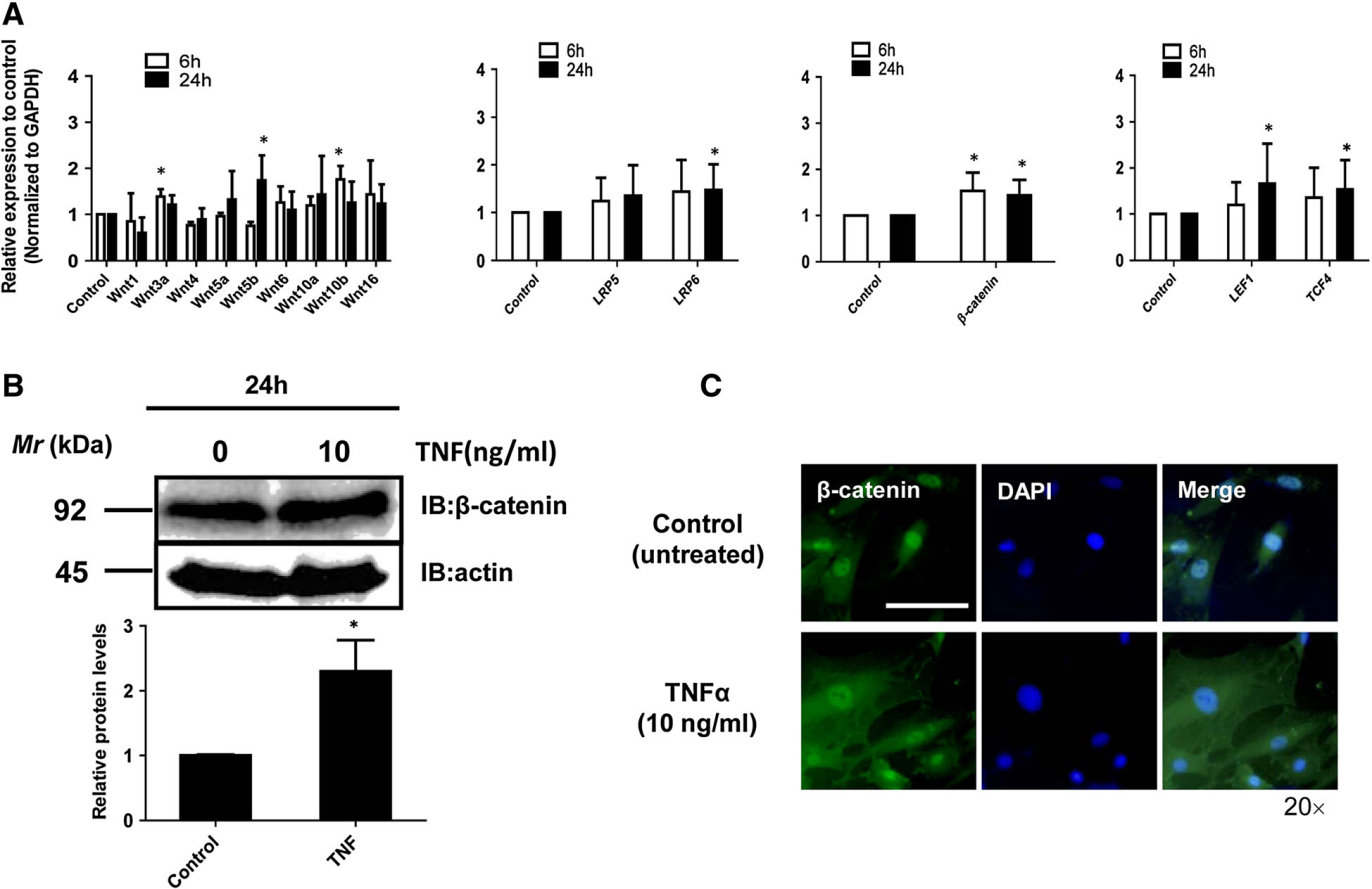
**TNF-α enhanced the expression of Wnt-related genes and proteins in nucleus pulposus cells. (A)** Wnt-related mRNA expression after exposure of nucleus pulposus cells to TNF-α (10 ng/mL) for 6 and 24 h, as assessed by real-time PCR. **(B)** Western blot analysis of β-catenin activation after treatment of nucleus pulposus cells with TNF-α (10 ng/mL). ^*^P < 0.05 indicates significant differences between groups. Error bars represent the SD. n.s., not significant. **(C)** Detection of β-catenin protein expression by immunofluorescence microscopy. Nucleus pulposus cells were cultured with or without 10 ng/mL TNF-α for 24 h, fixed, and stained with an antibody against c-fos. Left: cells stained with an antibody to β-catenin; middle: cells stained with 4′,6-diamidino-2-phenylindole (DAPI), to identify healthy nuclei; right: cells stained with an antibody to β-catenin and with DAPI. Scale bar, 50 μm (original magnification, 20×).

We examined further the expression of Wnt-related genes via real-time PCR after treatment with TNF-α (10 ng/mL) for 6 and 24 h. Real-time PCR analysis demonstrated that TNF-α treatment for 24 h increased the expression of the *Wnt5b*, *LRP6*, *LEF1*, and *TCF4* mRNA, but treatment for 6 h did not (Figure 5A). TNF-α significantly also increased the expression of *β-catenin* mRNA at 6 and 24 h. In addition, we determined the expression levels of the β-catenin protein in nucleus pulposus cells after treatment with TNF-α. Western blot analysis using an anti-β-catenin antibody demonstrated that TNF-α treatment increased the expression of the β-catenin protein (Figure 5B). Cells were evaluated by immunofluorescence analysis to determine whether a concomitant elevation in β-catenin protein expression was associated with the activation of TNF-α. Immunofluorescence analysis using an anti-β-catenin antibody showed that TNF-α treatment (10 ng/mL, 24 h) promoted the nuclear translocation of β-catenin more strongly in nucleus pulposus cells than in untreated control cells (Figure 5C).

## Discussion

The canonical Wnt signaling and the proinflammatory cytokine TNF-α play critical roles in development, homeostasis, and cancer [23-25]. However, the manner by which the Wnt signaling and TNF-α components interact in the complex network of biological communication that regulates these processes remains unclear. Our findings demonstrated for the first time that activation of Wnt signaling was regulated by the proinflammatory cytokine TNF-α. Our studies also revealed that Wnt signaling and TNF-α form a positive-feedback loop in nucleus pulposus cells. TNFs constitute a family of about 20 cytokines that bind to an increasing number of specific cell-surface receptors [26,27]. TNFs are produced as type II integral cell-surface proteins and exert their effects in a paracrine fashion by binding to, and inducing the trimerization of TNFRs. Through complex signaling networks, signal transducers lead to the activation of transcription factors, such as NF-κB, AP-1, and SP1, and their binding to the promoters of specific genes [28]. Regarding its role, TNF-α has been associated almost exclusively with inflammation or host defense. TNF-α was first described in IVDs in association with sciatic pain. Subsequent studies showed that TNF-α is widely expressed in humans with degenerative disc degeneration [29], as well as in animal models. Seguin *et al*. reported that TNF-α reduced the synthesis of matrix molecules and upregulated the mRNA expression of MMP-1, -3, and -13 and ADAM-TS4 and ADAM-TS5 [30]. Studies performed by Alsalameh and colleagues [31] on synovial fibroblasts from patients with rheumatoid arthritis and patients with osteoarthritis have also indicated there is a differential expression of the two TNF receptors (TNF-R1 and TNF-R2) in these cells and that, although both receptors can mediate the effect of TNF-α on TIMP1 expression, PGE_2_, the regulation of IL-6, and MMP-1 is mediated exclusively by TNF-R1.

Furthermore, Le Maitre *et al*. have shown that both IL-1 and TNF-α are expressed in IVDs and are upregulated in the presence of degeneration [16]. TNF-α can bind to, and signal through, either TNF-R1 or TNF-R2. Although TNF binds to each with high affinity, TNF-R1 is more ubiquitously expressed and it is generally believed that TNF-R1 is responsible for the majority of biological actions of TNF while TNF-R2 may function to potentiate the effects of TNF-R1. Freemont *et al*. have also reported that TNF-R2 is not expressed by IVD cells either in normal or degenerate IVDs [32]. In addition, they have demonstrated that human IVD cells are capable of responding to TNF-α *in vivo*. However, no increase in TNF-R1 synthesis was seen during IVD degeneration.

The complexity of the Wnt signaling cascade should enable the activation and/or repression of many specific signals and targets. Remarkably, we found that Wnt signaling, which suppresses the proliferation of nucleus pulposus cells and induces cell senescence, activated the expression of TNF-α. Direct evidence for the activation of TNF-α by Wnt signaling was obtained from experiments such as a reporter assay, real-time PCR, western blotting, et cetera. Furthermore, Wnt signaling was modulated by several different families of secreted negative regulators. The results of this study suggest that inhibition of Wnt signaling may be able to contribute to the suppression of disc degeneration, because Wnt signaling increased the expression of TNF-α and TNF-α activated Wnt signaling. Wnt antagonists can be divided into two functional classes. These two major classes function in quite different ways: the secreted Frizzled-related protein (sFRP) class binds to Wnt ligands, whereas the DKK or sclerostin class binds to a component of the Wnt receptor. Thus, in theory, the outcome appears to be that sFRPs inhibit canonical (β-catenin-dependent) and noncanonical (β-catenin-independent) pathways, whereas DKKs or sclerostin inhibit only the canonical pathway. The DKK family comprises four members (DKK-1 to DKK-4) in mammals [33,34]. DKK proteins have been implicated in various diseases, including retinal degeneration, malignancies, and cerebral ischemia [35-37].

The most-studied member of the DKK family is DKK-1. The binding of DKK-1 to the LPR5/6 receptor and a cell-surface coreceptor, Kremen-1/2, promotes internalization of the receptor complex and dampens the Wnt signal. Both DKK-1 and DKK-2 bind to the LRP5/6 receptor, which is expressed in cells and has higher affinity compared with Fz and Wnt. The characteristic developmental function of DKK-1 is its head-inducing activity. Previously, Ye *et al*. reported that TNF-α increased the expression of β-catenin and MMP-13, and significantly inhibited matrix synthesis, which resulted in the degeneration of rabbit IVD cells. These authors also showed that blocking Wnt/β-catenin signaling using DKK-1 protected the normal metabolism of IVD tissues in rabbits [38]. However, the present study showed an absence of changes in TNF-α promoter activity when the DKK-1 plasmid was used. These differences may be related to the age and species of the animal from which the cells were isolated, and the environment in which the cell metabolism is studied.

The Wnt-antagonizing activity of DKK-4 appears to be indistinguishable from that of DKK-1, whereas DKK-3 has distinct roles in regulating the Wnt pathway, depending on the cell types examined. For example, DKK-3 potentiates Wnt signaling in human Müller glia MIO-M1 and HEK293 cell lines [39], but inhibits Wnt signaling in PC12 and osteocarcinoma Saos-2 cells [40,41]. The biological roles of DKK-3 in Wnt signaling remain unclear, because it does not inhibit canonical Wnt signaling [33,42]. In addition, DKK-3 does not interact with LRPs or Krm1/2 [42,43]. It also remains to be determined whether DKK-3 antagonizes other growth-factor pathways via mechanisms that involve a direct association with ligands or transmembrane receptors in a manner similar to that by which DKK-1 inhibits Wnt signaling. However, recent studies have revealed that the effect of DKK antagonists is not quite so simple. In fact, it may turn out that certain antagonists act as such only when expressed at nonphysiological levels. Therefore, there are clearly many unresolved issues regarding this subject. Furthermore, we checked the effect of sclerostin, which is a different inhibitor of Wnt signaling, on TNF-α promoter activity in nucleus pulposus cells. The results of this experiment showed significant inhibition of the TNF-α promoter after treatment with high sclerostin. Sclerostin is the product of the *SOST* gene. Similar to DKK, sclerostin binds to Lrp5/6 and antagonizes canonical Wnt signaling [44-46]. Low sclerostin expression leads to bone growth, whereas high expression inhibits bone formation. Recently, TNF has been identified as an inducer of sclerostin expression, but the current study showed that TNF-α suppressed the *SOST* gene. As a result, it might have resulted in the activation of Wnt signaling in nucleus pulposus cells.

Overall, our results indicate that inhibition of Wnt signaling suppresses a catabolic response via the inhibitory action of TNF-α in nucleus pulposus cells. However, it remains to be determined which of the proposed receptors for DKKs or sclerostin, including LRP-4, -5, and -6, are important for the responses observed in our study and whether the effect is dependent on canonical or noncanonical Wnt signaling, or on other types of signaling. In addition to the limitation inherent in the use of rats, because of the ambiguity of the notochordal cells in this animal, the functions of the rat disc compared with the functions of the human IVD need to be considered in the interpretation of the findings of this study. Regarding this issue, additional studies using different species are required to evaluate and conclude whether the mechanism involving the expression of these molecules is specific to nucleus pulposus cells, in particular regarding the human situation.

## Conclusions

Here, we have demonstrated that Wnt signaling regulates TNF-α and that Wnt signaling and TNF-α form a positive-feedback loop in nucleus pulposus cells. The results of the present study provide *in vitro* evidence that the activation of Wnt signaling upregulates the proinflammatory cytokine TNF-α and might cause the degeneration of nucleus pulposus cells. We speculated that blocking the Wnt signaling might protect nucleus pulposus cells against degeneration. The DKK or sclerostin families of proteins are natural regulators of Wnt signaling and can specifically block this pathway. These findings suggest that overexpression of DKK-3, DKK-4, or sclerostin inhibit TNF-α expression by specifically blocking the Wnt channel. Inhibition of Wnt signaling using DKKs or sclerostin exerts a protective and reversing effect in the TNF-α-induced degeneration of IVD cells.

## Abbreviations

ADAMTS: A disintegrin and metalloproteinase with thrombospondin motifs; BIO: 6-bromoindirubin-3′-oxime; BSA: Bovine serum albumin; Ct: Intensity threshold; DAPI: 4′,6-diamidino-2-phenylindole; DKK: Dickkopf; DMEM: Dulbecco’s modified Eagle’s medium; FBS: Fetal bovine serum; GAPDH: Glyceraldehyde 3-phosphate dehydrogenase; GSK: Glycogen synthase kinase; IL-1β: Interleukin-1 β; IVD: Intervertebral disc; LEF: Lymphoid enhancer factor; MMP: Matrix metalloproteinase; PBS: Phosphate-buffered saline; PCP: Planar cell polarity; PGE2: Prostaglandin E_2_; RT-PCR: Reverse transcription polymerase chain reaction; sFRP: Frizzled-related protein; SOST: Sclerostin; TBST: Tris-buffered saline and Tween 20; TCF: T-cell factor; TNF-α: Tumor necrosis factor-α; WT: Wild-type.

## Competing interests

The authors declare that they have no competing interests.

## Authors’ contributions

AH participated in study design, performed experiments, analyzed data and wrote the paper. KY prepared the isolation of cells and performed experiments and the statistical analysis. TN performed experiments and analyzed data. DS participated in study design, performed experiments and performed the statistical analysis. JM participated in study design and coordination and helped to draft the manuscript. All authors read and approved the final manuscript.

## Supplementary Material

Additional file 1: Figure S1 The soluble TNF-α protein levels after the stimulation of TNF-α (10 ng/mL, 24 h) by western blotting. The results demonstrate that soluble TNF-α protein was significantly elevated after the stimulation of TNF-α compared to the control cells. Click here for file

## References

[B1] Hiyama A Sakai D Tanaka M Arai F Nakajima D Abe K Mochida J The relationship between the Wnt/beta-catenin and TGF-beta/BMP signals in the intervertebral disc cell J Cell Physiol 2011 15 1139 1148 10.1002/jcp.22438 20945354

[B2] Hiyama A Sakai D Arai F Nakajima D Yokoyama K Mochida J Effects of a glycogen synthase kinase-3beta inhibitor (LiCl) on c-myc protein in intervertebral disc cells J Cell Biochem 2011 15 2974 2986 10.1002/jcb.23217 21678465

[B3] Hiyama A Sakai D Risbud MV Tanaka M Arai F Abe K Mochida J Enhancement of intervertebral disc cell senescence by WNT/beta-catenin signaling-induced matrix metalloproteinase expression Arthritis Rheum 2010 15 3036 3047 10.1002/art.27599 20533544 PMC3622204

[B4] Wang M Tang D Shu B Wang B Jin H Hao S Dresser KA Shen J Im HJ Sampson ER Rubery PT Zuscik MJ Schwarz EM O'Keefe RJ Wang Y Chen D Conditional activation of beta-catenin signaling in mice leads to severe defects in intervertebral disc tissue Arthritis Rheum 2012 15 2611 2623 10.1002/art.34469 22422036 PMC3632450

[B5] Slusarski DC Yang-Snyder J Busa WB Moon RT Modulation of embryonic intracellular Ca2+ signaling by Wnt-5A Dev Biol 1997 15 114 120 10.1006/dbio.1996.8463 9073455

[B6] Tree DR Ma D Axelrod JD A three-tiered mechanism for regulation of planar cell polarity Semin Cell Dev Biol 2002 15 217 224 10.1016/S1084-9521(02)00042-3 12137730

[B7] Yamaguchi TP Heads or tails: Wnts and anterior-posterior patterning Curr Biol 2001 15 R713 R724 10.1016/S0960-9822(01)00417-1 11553348

[B8] Lee E Salic A Kruger R Heinrich R Kirschner MW The roles of APC and Axin derived from experimental and theoretical analysis of the Wnt pathway PLoS Biol 2003 15 E10 10.1371/journal.pbio.0000010 14551908 PMC212691

[B9] Giles RH van Es JH Clevers H Caught up in a Wnt storm: Wnt signaling in cancer Biochim Biophys Acta 2003 15 1 24 12781368 10.1016/s0304-419x(03)00005-2

[B10] Church VL Francis-West P Wnt signalling during limb development Int J Dev Biol 2002 15 927 936 12455630

[B11] He TC Sparks AB Rago C Hermeking H Zawel L da Costa LT Morin PJ Vogelstein B Kinzler KW Identification of c-MYC as a target of the APC pathway Science 1998 15 1509 1512 9727977 10.1126/science.281.5382.1509

[B12] Hoy D Brooks P Blyth F Buchbinder R The epidemiology of low back pain Best Pract Res Clin Rheumatol 2010 15 769 781 10.1016/j.berh.2010.10.002 21665125

[B13] Juniper M Le TK Mladsi D The epidemiology, economic burden, and pharmacological treatment of chronic low back pain in France, Germany, Italy, Spain and the UK: a literature-based review Expert Opin Pharmacother 2009 15 2581 2592 10.1517/14656560903304063 19874246

[B14] Hunter CJ Matyas JR Duncan NA The three-dimensional architecture of the notochordal nucleus pulposus: novel observations on cell structures in the canine intervertebral disc J Anat 2003 15 279 291 10.1046/j.1469-7580.2003.00162.x 12713268 PMC1571084

[B15] Hunter CJ Matyas JR Duncan NA Cytomorphology of notochordal and chondrocytic cells from the nucleus pulposus: a species comparison J Anat 2004 15 357 362 10.1111/j.0021-8782.2004.00352.x 15575884 PMC1571361

[B16] Le Maitre CL Hoyland JA Freemont AJ Catabolic cytokine expression in degenerate and herniated human intervertebral discs: IL-1beta and TNFalpha expression profile Arthritis Res Ther 2007 15 R77 10.1186/ar2275 17688691 PMC2206382

[B17] Burke JG Watson RW McCormack D Dowling FE Walsh MG Fitzpatrick JM Intervertebral discs which cause low back pain secrete high levels of proinflammatory mediators J Bone Joint Surg Br 2002 15 196 201 10.1302/0301-620X.84B2.12511 11924650

[B18] Legendre F Dudhia J Pujol JP Bogdanowicz P JAK/STAT but not ERK1/ERK2 pathway mediates interleukin (IL)-6/soluble IL-6R down-regulation of Type II collagen, aggrecan core, and link protein transcription in articular chondrocytes. Association with a down-regulation of SOX9 expression J Biol Chem 2003 15 2903 2912 10.1074/jbc.M110773200 12419823

[B19] Legendre F Bogdanowicz P Boumediene K Pujol JP Role of interleukin 6 (IL-6)/IL-6R-induced signal tranducers and activators of transcription and mitogen-activated protein kinase/extracellular J Rheumatol 2005 15 1307 1316 15996070

[B20] Lee JM Song JY Baek M Jung HY Kang H Han IB Kwon YD Shin DE Interleukin-1beta induces angiogenesis and innervation in human intervertebral disc degeneration J Orthop Res 2011 15 265 269 10.1002/jor.21210 20690185

[B21] Fujita K Janz S Attenuation of WNT signaling by DKK-1 and -2 regulates BMP2-induced osteoblast differentiation and expression of OPG RANKL and M-CSF. Mol Cancer 2007 15 71 10.1186/1476-4598-6-71 PMC2173906 17971207

[B22] Sato N Meijer L Skaltsounis L Greengard P Brivanlou AH Maintenance of pluripotency in human and mouse embryonic stem cells through activation of Wnt signaling by a pharmacological GSK-3-specific inhibitor Nature Med 2004 15 55 63 10.1038/nm979 14702635

[B23] Naugler WE Karin M NF-kappaB and cancer-identifying targets and mechanisms Curr Opin Genet Dev 2008 15 19 26 10.1016/j.gde.2008.01.020 18440219 PMC2587362

[B24] Courtois G The NF-kappaB signaling pathway in human genetic diseases Cell Mol Life Sci 2005 15 1682 1691 10.1007/s00018-005-5031-5 15924263 PMC11139074

[B25] Clevers H Wnt/beta-catenin signaling in development and disease Cell 2006 15 469 480 10.1016/j.cell.2006.10.018 17081971

[B26] Baker SJ Reddy EP Modulation of life and death by the TNF receptor superfamily Oncogene 1998 15 3261 3270 9916988 10.1038/sj.onc.1202568

[B27] Gruss HJ Dower SK Tumor necrosis factor ligand superfamily: involvement in the pathology of malignant lymphomas Blood 1995 15 3378 3404 7780126

[B28] Baud V Karin M Signal transduction by tumor necrosis factor and its relatives Trends Cell Biol 2001 15 372 377 10.1016/S0962-8924(01)02064-5 11514191

[B29] Weiler C Nerlich AG Bachmeier BE Boos N Expression and distribution of tumor necrosis factor alpha in human lumbar intervertebral discs: a study in surgical specimen and autopsy controls Spine (Phila Pa 1976) 2005 15 44 53 discussion 54 10.1097/01.brs.0000174529.07959.c0 15626980

[B30] Seguin CA Pilliar RM Roughley PJ Kandel RA Tumor necrosis factor-alpha modulates matrix production and catabolism in nucleus pulposus tissue Spine (Phila Pa 1976) 2005 15 1940 1948 10.1097/01.brs.0000176188.40263.f9 16135983

[B31] Alsalameh S Amin RJ Kunisch E Jasin HE Kinne RW Preferential induction of prodestructive matrix metalloproteinase-1 and proinflammatory interleukin 6 and prostaglandin E2 in rheumatoid arthritis synovial fibroblasts via tumor necrosis factor receptor-55 J Rheumatol 2003 15 1680 1690 12913922

[B32] Freemont AJ Watkins A Le Maitre C Baird P Jeziorska M Knight MT Ross ER O’Brien JP Hoyland JA Nerve growth factor expression and innervation of the painful intervertebral disc J Pathol 2002 15 286 292 10.1002/path.1108 12115873

[B33] Krupnik VE Sharp JD Jiang C Robison K Chickering TW Amaravadi L Brown DE Guyot D Mays G Leiby K Chang B Duong T Goodearl AD Gearing DP Sokol SY McCarthy SA Functional and structural diversity of the human Dickkopf gene family Gene 1999 15 301 313 10.1016/S0378-1119(99)00365-0 10570958

[B34] Monaghan AP Kioschis P Wu W Zuniga A Bock D Poustka A Delius H Niehrs C Dickkopf genes are co-ordinately expressed in mesodermal lineages Mech Dev 1999 15 45 56 10.1016/S0925-4773(99)00138-0 10495270

[B35] Mastroiacovo F Busceti CL Biagioni F Moyanova SG Meisler MH Battaglia G Caricasole A Bruno V Nicoletti F Induction of the Wnt antagonist, Dickkopf-1, contributes to the development of neuronal death in models of brain focal ischemia J Cereb Blood Flow Metab 2009 15 264 276 10.1038/jcbfm.2008.111 18827832

[B36] Hsieh SY Hsieh PS Chiu CT Chen WY Dickkopf-3/REIC functions as a suppressor gene of tumor growth Oncogene 2004 15 9183 9189 15516983 10.1038/sj.onc.1208138

[B37] Hackam AS Strom R Liu D Qian J Wang C Otteson D Gunatilaka T Farkas RH Chowers I Kageyama M Leveillard T Sahel JA Campochiaro PA Parmigiani G Zack DJ Identification of gene expression changes associated with the progression of retinal degeneration in the rd1 mouse Invest Ophthalmol Vis Sci 2004 15 2929 2942 10.1167/iovs.03-1184 15326104

[B38] Ye S Wang J Yang S Xu W Xie M Han K Zhang B Wu Z Specific inhibitory protein Dkk-1 blocking Wnt/beta-catenin signaling pathway improve protectives effect on the extracellular matrix J Huazhong Univ Sci Technolog Med Sci 2011 15 657 662 10.1007/s11596-011-0577-y 22038356

[B39] Nakamura RE Hunter DD Yi H Brunken WJ Hackam AS Identification of two novel activities of the Wnt signaling regulator Dickkopf 3 and characterization of its expression in the mouse retina BMC Cell Biol 2007 15 52 10.1186/1471-2121-8-52 18093317 PMC2233618

[B40] Hoang BH Kubo T Healey JH Yang R Nathan SS Kolb EA Mazza B Meyers PA Gorlick R Dickkopf 3 inhibits invasion and motility of Saos-2 osteosarcoma cells by modulating the Wnt-beta-catenin pathway Cancer Res 2004 15 2734 2739 10.1158/0008-5472.CAN-03-1952 15087387

[B41] Caricasole A Ferraro T Iacovelli L Barletta E Caruso A Melchiorri D Terstappen GC Nicoletti F Functional characterization of WNT7A signaling in PC12 cells: interaction with A FZD5 x LRP6 receptor complex and modulation by Dickkopf proteins J Biol Chem 2003 15 37024 37031 10.1074/jbc.M300191200 12857724

[B42] Mao B Wu W Li Y Hoppe D Stannek P Glinka A Niehrs C LDL-receptor-related protein 6 is a receptor for Dickkopf proteins Nature 2001 15 321 325 10.1038/35077108 11357136

[B43] Mao B Wu W Davidson G Marhold J Li M Mechler BM Delius H Hoppe D Stannek P Walter C Glinka A Niehrs C Kremen proteins are Dickkopf receptors that regulate Wnt/beta-catenin signalling Nature 2002 15 664 667 10.1038/nature756 12050670

[B44] Li X Zhang Y Kang H Liu W Liu P Zhang J Harris SE Wu D Sclerostin binds to LRP5/6 and antagonizes canonical Wnt signaling J Biol Chem 2005 15 19883 19887 10.1074/jbc.M413274200 15778503

[B45] Ellies DL Viviano B McCarthy J Rey JP Itasaki N Saunders S Krumlauf R Bone density ligand, Sclerostin, directly interacts with LRP5 but not LRP5G171V to modulate Wnt activity J Bone Miner Res 2006 15 1738 1749 10.1359/jbmr.060810 17002572

[B46] Semenov M Tamai K He X SOST is a ligand for LRP5/LRP6 and a Wnt signaling inhibitor J Biol Chem 2005 15 26770 26775 10.1074/jbc.M504308200 15908424

