# Supplementary figures and images for: A complex interaction between Wnt signaling and TNF-α in nucleus pulposus cells
Source: Arthritis Res Ther. 2013 Nov 14;15(6):R189. doi: 10.1186/ar4379 (PMC3978705; doi:10.1186/ar4379)

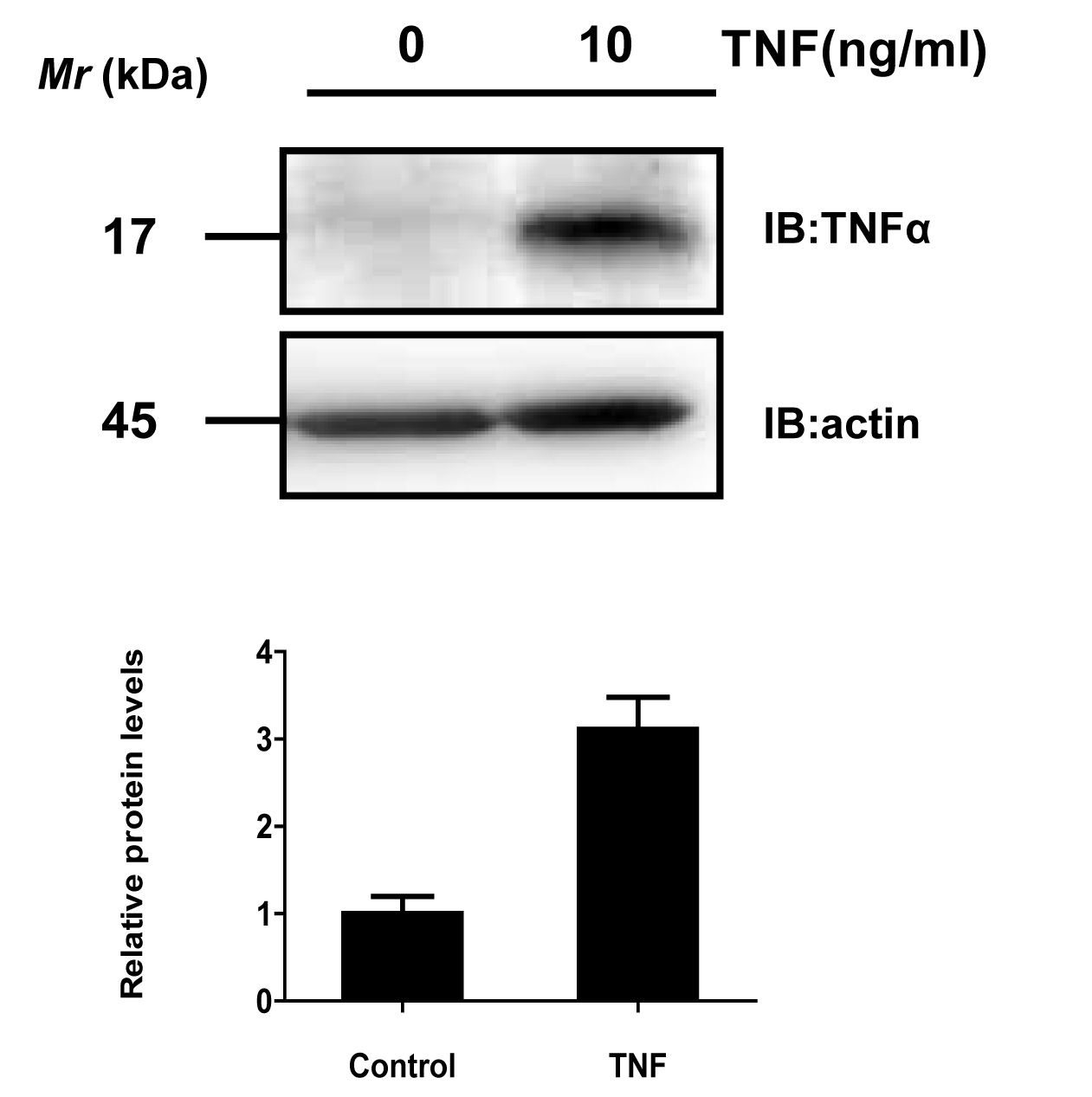

Supplement: Additional file 1: Figure S1 — The soluble TNF-α protein levels after the stimulation of TNF-α (10 ng/mL, 24 h) by western blotting. The results demonstrate that soluble TNF-α protein was significantly elevated after the stimulation of TNF-α compared to the control cells. [file ar4379-S1.jpeg]
